# Melanoma Stem Cells Educate Neutrophils to Support Cancer Progression

**DOI:** 10.3390/cancers14143391

**Published:** 2022-07-13

**Authors:** Martina Anselmi, Fabrizio Fontana, Monica Marzagalli, Nicoletta Gagliano, Michele Sommariva, Patrizia Limonta

**Affiliations:** 1 Department of Pharmacological and Biomolecular Sciences, Università degli Studi di Milano, 20133 Milano, Italy; martina.anselmi@unimi.it (M.A.); monica.marzagalli@unimi.it (M.M.); 2 Department of Biomedical Sciences for Health, Università degli Studi di Milano, 20133 Milano, Italy; nicoletta.gagliano@unimi.it (N.G.); michele.sommariva@unimi.it (M.S.)

**Keywords:** melanoma, cancer stem cells, neutrophils, cytokines, ROS, NETs

## Abstract

**Simple Summary:**

In melanoma patients, poor prognosis often correlates with high presence of cancer-associated neutrophils, indicating that tumors can recruit these immune cells to specifically sustain their own development and progression. However, the role of cancer stem cells (CSCs) in this dialogue has not been elucidated yet. Our results revealed that melanoma SCs can reshape the immune microenvironment by triggering a pro-tumor N2 phenotype in neutrophils, which in turn are able to confer stemness properties to melanoma cells.

**Abstract:**

Background: It is now well-established that cancer stem cells (CSCs) can support melanoma progression by reshaping the tumor immune microenvironment. However, the molecular mechanisms underlying the crosstalk between melanoma SCs and cancer-associated neutrophils have not been elucidated yet. Methods: The aim of the present study was to unravel the role of melanoma SCs in neutrophil polarization. HL60 neutrophil-like (dHL60) cells were treated with conditioned medium from A375 melanoma SCs (CSC-CM), and their phenotype was investigated. Results: We demonstrated that CSC-CM could specifically activate immune cells by increasing CD66b and CD11b expression. In particular, we revealed that A375 CSCs could release various soluble factors, namely TGF-β, IL-6, and IL-8, able to promote the recruitment of neutrophils and their switch toward an N2 phenotype characterized by the activation of ERK, STAT3, and P38 pathways and the overexpression of CXCR2 and NF-kB. Moreover, after exposure to CSC-CM, dHL60 cells exhibited enhanced ROS production and NET release, without undergoing cell death; increased secretion of MMP-9 and pro-inflammatory cytokines was also observed. Finally, CSC-CM-activated neutrophils endowed A375 cells with stemness traits, stimulating both sphere formation and ABCG2 expression. Conclusion: Collectively, our results suggest that melanoma SCs can prime neutrophils to support cancer progression.

## 1. Introduction

Despite accounting for only 5% of skin cancers, cutaneous melanoma represents the deadliest tumor in this category, showing a mortality rate of 75% [1]. Notably, patient survival directly correlates with early detection, with late-stage malignancies exhibiting a very poor prognosis [2]. In this setting, promising therapeutic approaches, including immunotherapy, have been recently developed; however, tumor relapse and the insurgence of drug resistance remain fundamental unsolved problems.

Increasing evidence has revealed that cancer stem cells (CSCs), a subpopulation of tumor cells characterized by self-renewal ability, are responsible for disease progression, metastasis, and reduced response to anti-neoplastic treatments [3,4]. Their presence in melanoma tissues and their role in tumor recurrence are well-documented [5]. Nonetheless, little is known about their interplay with the tumor microenvironment (TME).

Neutrophils are the most abundant immune cells in TME. Classically, they represent the first line of defense in the innate arm of the immune system; however, various pro-inflammatory cancer-derived cytokines, such as transforming growth factor-beta (TGF-β), interleukin 6 (IL-6), and interleukin 8 (IL-8), have been reported to drive neutrophil recruitment and polarization, promoting their switch from an anti-tumor (N1) to an immunosuppressive and tumor-sustaining phenotype (N2) [6,7]. In particular, while the N1 profile is characterized by reduced proliferation and by the secretion of high levels of tumor necrosis factor-alpha (TNF-α), N2 cells are long-living and can favor cancer development by releasing specific pro-tumor factors, such as reactive oxygen species (ROS), neutrophil extracellular traps (NETs), IL-6, IL-8, and matrix metalloproteinase 9 (MMP-9) [8]. In this regard, it should be noted that in melanoma patients, a high presence of cancer-associated neutrophils correlates with limited therapeutic outcomes [9]. As mentioned above, while the existence of a bidirectional dialogue between these cells and CSCs has been highlighted in colorectal carcinoma and glioma [10,11], the complex interaction network occurring in the melanoma immune microenvironment still needs to be elucidated.

Herein, we dissected the molecular mechanisms underlying the bidirectional crosstalk between melanoma SCs and neutrophils, with a focus on the role of the first in educating the immune system to support tumor progression.

## 2. Materials and Methods

### 2.1. Chemicals

The following primary antibodies were utilized for Western blot analyses: ABCG2 (B1) was from Santa Cruz Biotechnology Inc. (Dallas, TX, USA); p-ERK (2938), ERK (4695), p-STAT3 (9145), STAT3 (4904), P38 (8690), p-P38 (4511), NF-kB (8242), GADPH (5174) were from Cell Signaling Technology Inc. (Danvers, MA, USA). All the antibodies were used at the concentration 1:1000. HRP-conjugated secondary antibodies were from Cell Signaling Technology Inc. and enhanced chemiluminescence reagents were from Cyanagen (Bologna, Italy).

Regarding flow cytometry analyses, CD11b (12-0118-41), CD66b (17-066-42), and CXCR2 (12-1829-42) were from Invitrogen Inc. (Waltham, MA, USA).

EGF, FGF, and N2 were from Thermo Fisher Scientific (Waltham, MA, USA). Dimethyl sulfoxide (DMSO) was from Sigma-Aldrich (Milano, Italy).

### 2.2. Cell Lines and Cell Cultures

A375 human melanoma cells and HL60 human promyelocytic leukemia cells were from American Type Culture Collection (ATCC, Manassas, VA, USA). A375 cells were cultured in DMEM supplemented with 10% FBS, glutamine (1 mmol/L), and antibiotics (100 IU/mL penicillin G sodium and 100 μg/mL streptomycin sulfate). HL60 cells were grown in IMDM supplemented with 20% FBS, glutamine (1 mmol/L), HEPES (5 mM), and antibiotics (100 IU/mL penicillin G sodium and 100 μg/mL streptomycin sulfate) and were induced to differentiate into neutrophil-like (dHL60) cells by treatment with 1.3% (*v/v*) DMSO for 72 h.

Melanospheres were obtained and passaged as previously described [12].

All the cells were recovered from storage in liquid nitrogen and cultured in humidified atmosphere of 5% CO_2_/95% air at 37 °C for no more than 10–12 weeks.

### 2.3. Conditioned Medium (CM) Preparation

A375 CSCs were seeded in 25-cm^2^ flasks at a density of 8 × 10^5^ cells for 24 h in melanosphere-conditioned medium mixed with fresh Euromed-N (1:3 ratio). The supernatant was then collected, and cell debris was removed by centrifugation at 4400 rpm for 3 min and stored at −20 °C until use.

The dHL60 cells were seeded in a 25-cm^2^ flask at a density of 1 × 10^5^ cells and treated with CSC–CM for 4 and 24 h, then washed with PBS and cultured in serum-free medium for 24 h (dHL60–CM). The dHL60–CM was collected and stored at −20 °C.

### 2.4. CD11b, CD66b, and IL-8 Receptor (CXCR2) Expression

In dHL60 cells, CD11b, CD66b, and CXCR2 expression was assessed by flow cytometry. Cells were seeded in six well-plates (1 × 10^5^ cells/well) and conditioned with CSC–CM for 1 h, then resuspended in ice-cold PBS containing 2% FBS and incubated with the specific antibodies for 30 min. Finally, they were analyzed with a Novocyte3000 instrument (ACEA Biosciences, San Diego, CA, USA). Data analysis was conducted with Novoexpress software.

### 2.5. Chemotaxis Assay

Chemotaxis assay was performed using transwell filters (8 μm pore size). Briefly, 1 × 10^5^ cells/well were placed in the top chambers of a 24-well plate, in which the bottom chambers were filled with CSC–CM. After incubation for 3 h at 37 °C, cells that migrated to the lower chamber were stained with DiffQuick staining kit (DADE, Dudingen, Switzerland) and counted.

### 2.6. ROS Detection

To determine intracellular ROS production, dHL60 cells were seeded in six well-plates (1 × 10^5^ cells/well) in the presence of CSC–CM. After 4 h, they were incubated with 2′,7′-dichlorofluorescin diacetate (Sigma Aldrich, St. Louis, MO, USA) 10 μM for 30 min, and fluorescence was measured by flow cytometry, using a Novocyte3000 instrument and Novoexpress software.

To determine extracellular ROS production, dHL60 cells were seeded in 96 well-plates (1 × 10^4^ cells/well) in the presence of CSC–CM. After 4 h, they were incubated with 2′,7′-dichlorofluorescin diacetate (Sigma Aldrich) 10 μM for 30 min, and fluorescence was measured by EnSpire Multimode Plate reader (PerkinElmer Waltham, MA, USA).

### 2.7. NET Release

To investigate the induction of NETs, dHL60 cells were seeded in 24 well-plates (1 × 10^5^ cells/well) and conditioned with CSC–CM for 4 h. Then, they were fixed with 4% PAF and incubated with SYTOX green (Invitrogen Inc.) in PBS for 10 min. Labeled cells were examined under a Zeiss Axiovert 200 microscope with a 63 × 1.4 objective lens linked to a Coolsnap Es CCD camera (Roper Scientific-Crisel Instruments, Roma, Italy).

### 2.8. Annexin V/PI Apoptosis Assay

dHL60 cells were seeded in six well-plates (1 × 10^5^ cells/well) and treated with CSC–CM for 4 and 24 h. Adherent and floating cells were then harvested, washed in PBS, and incubated with Annexin V and PI, using the eBioscience™ Annexin V-FITC Apoptosis Detection Kit. Finally, they were analyzed with a Novocyte3000 instrument. Data analysis was conducted with Novoexpress software.

### 2.9. ELISA Assay

The Elisa assay kits for evaluating IL-6 (DY206-05), IL-8 (DY208-05), TNF-α (DY210-05), TGF-β (DY240-05), MMP-9 (DY911-05) secretion in CSC–CM and/or dHL60–CM were from R&D Systems (Minneapolis, MN, USA). Absorbance was measured by EnSpire Multimode Plate reader.

### 2.10. Melanosphere Formation Assay

Adherent A375 cells were seeded in 25-cm^2^ flasks (5 × 10^5^ cell/flask) and incubated with dHL60–CM for 7 days to determine their spheroidogenic potential. Melanospheres were photographed and counted with Zeiss Axiovert 200 microscope with a 20 × 1.4 objective lens linked to a Coolsnap Es CCD camera.

### 2.11. Western Blot Analysis

dHL60 cells were seeded at 1 × 10^5^ cells in 25-cm^2^ flasks and incubated with CSC–CM for 4 h. Then, they were lysed in RIPA buffer; protein extracts (20 μg) were resolved on SDS-PAGE and transferred to nitrocellulose membranes (Biorad, Hercules, CA, USA), which were exposed to the specific primary and HRP-conjugated secondary antibodies. Enhanced chemiluminescence was produced by using Westar Etac Ultra 2.0, XLS075,0100. GAPDH was used as a loading control. The original WB can be found in Supplementary Materials.

### 2.12. Statistical Analysis

A statistics package (GraphPad Prism5, GraphPad Software San Diego, CA, USA) was used for statistical analysis. Data are expressed as the mean ± SEM of three independent experiments. Differences between groups were evaluated by *t*-test. A *p* value < 0.05 was considered statistically significant.

## 3. Results

### 3.1. Melanoma SC–CM Promotes the Activation of Neutrophils

**Figure 1:**
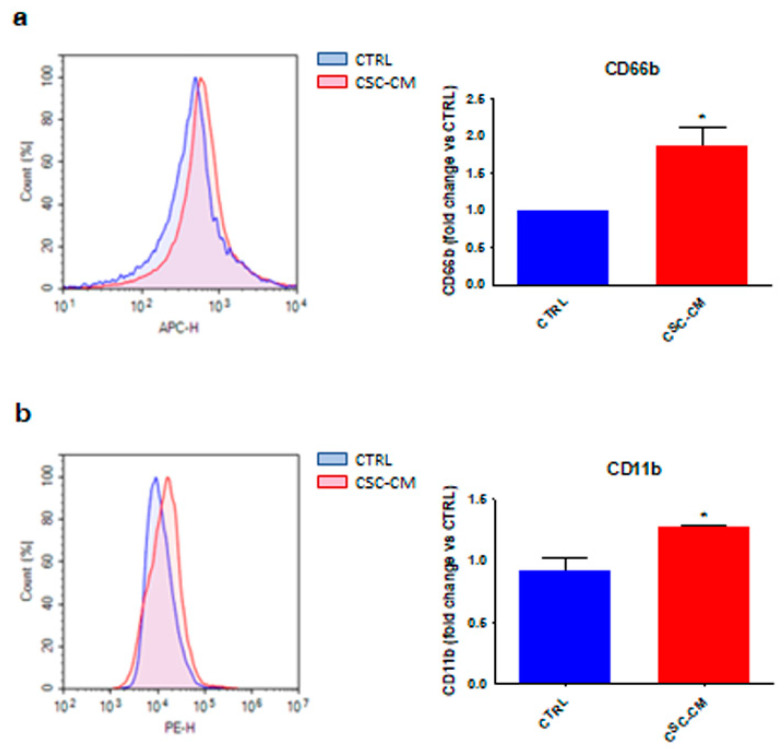
Melanoma SC–CM promotes the activation of neutrophils. (**a**) dHL60 cells were incubated with CSC–CM for 1 h. The expression of CD66b was evaluated by flow cytometry. Each experiment was repeated three times. Data are expressed as mean values ± SEM and were analyzed by *t*-test. * *p* < 0.05 vs. ctrl (control). (**b**) dHL60 cells were incubated with CSC–CM for 1 h. The expression of CD11b was evaluated by flow cytometry. Each experiment was repeated three times. Data are expressed as mean values ± SEM and were analyzed by *t*-test. * *p* < 0.05 vs. ctrl (control).

To investigate the effects of melanoma SCs on neutrophils, dHL60 cells were incubated with A375 CSC–CM. Interestingly, this resulted in an increased expression of CD66b and CD11b (Figure 1a,b), two well-known surface markers correlating with an active/mature immune state [13], suggesting that dHL60 cells can be activated by CSC–CM.

### 3.2. Melanoma SCs Release Key Factors Responsible for Neutrophil Recruitment and N1-to-N2 Phenotype Reprogramming

**Figure 2:**
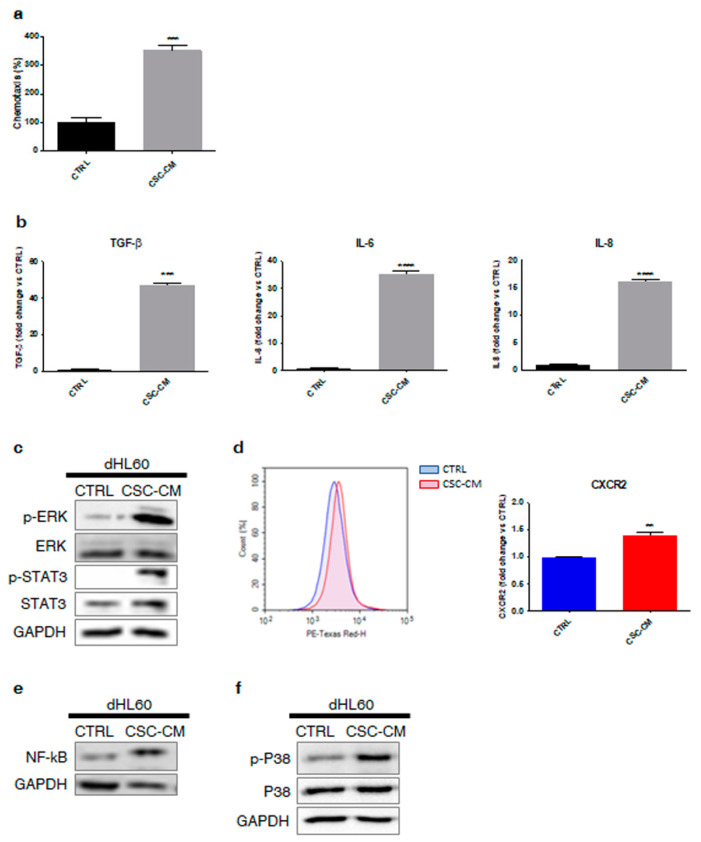
Melanoma SCs release key factors responsible for neutrophil recruitment and N1-to-N2 phenotype reprogramming. (**a**) Chemotaxis assay was performed by exposing dHL60 cells to CSC–CM for 3 h. Each experiment was repeated three times. Data are expressed as mean values ± SEM and were analyzed by *t*-test. *** *p* < 0.001 vs. ctrl (control). (**b**) dHL60 cells were incubated with CSC–CM for 4 h. TGF-β, IL-6, and IL-8 secretion was then assessed by Elisa assay. Each experiment was repeated three times. Data are expressed as mean values ± SEM and were analyzed by *t*-test. *** *p* < 0.001 vs. ctrl (control), **** *p* < 0.0001 vs. ctrl (control). (**c**) dHL60 cells were incubated with CSC–CM for 4 h. Western blot analysis was carried out to analyze the expression of p-STAT3 and p-ERK. GAPDH was used as a loading control. One representative experiment from three independent tests is shown. (**d**) dHL60 cells were incubated with CSC–CM for 1 h. The expression of CXCR2 was then evaluated by flow cytometry. Each experiment was repeated three times. Data are expressed as mean values ± SEM and were analyzed by *t*-test. ** *p* < 0.01 vs. ctrl (control). (**e**) dHL60 cells were incubated with CSC–CM for 4 h. Western blot analysis was carried out to analyze the expression of NF-kB. GAPDH was used as a loading control. One representative experiment of three independent tests is shown. (**f**) dHL60 cells were incubated with CSC–CM for 4 h. Western blot analysis was carried out to analyze the expression of p-P38. GAPDH was used as a loading control. One representative experiment of three independent tests is shown.

Based on the above findings, we hypothesized that melanoma SCs were able to recruit neutrophils, endowing them with N2 properties. To confirm this supposition, we examined the effects of A375 CSC–CM on dHL60 cell line, highlighting a higher chemotactic activity in treated cells (Figure 2a). Then, we analyzed the molecular content of A375 CSC–CM, which was found to be enriched in pro-inflammatory cytokines commonly involved in neutrophil recruitment and phenotypic switch from N1 to N2, including TGF-β, IL-6, and IL-8 (Figure 2b). Notably, upregulation of STAT3 and ERK pathways, two IL-6-associated cascades, and enhanced expression of the IL-8 receptor CXCR2 and of its downstream target NF-kB were observed in immune cells exposed to CSC–CM (Figure 2c–e); likewise, phosphorylation of P38, a well-known molecular cascade correlating with neutrophil chemotaxis and N2 polarization [14,15,16], was evidenced (Figure 2f). Overall, these results indicate that melanoma SCs can produce important soluble factors able to drive the transition of neutrophils into an N2 phenotype.

### 3.3. Melanoma SC–CM-Treated Neutrophils Exhibit Increased Production of Both Intracellular and Extracellular ROS

**Figure 3:**
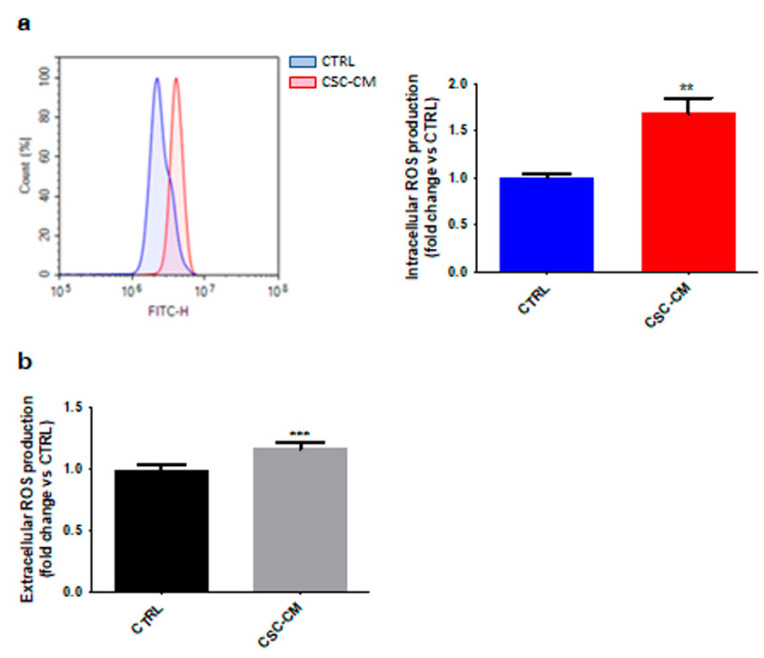
Melanoma SC–CM-treated neutrophils exhibit increased production of both intracellular and extracellular ROS. (**a**) dHL60 cells were incubated with CSC–CM for 4 h. Intracellular ROS production was then measured by flow cytometry after staining with 2′,7′-dichlorofluorescin diacetate. Each experiment was repeated three times. Data are expressed as mean values ± SEM and were analyzed by *t*-test. ** *p* < 0.01 vs. ctrl (control). (**b**) dHL60 cells were incubated with CSC–CM for 4 h. Extracellular ROS production was then measured by fluorescence analysis after staining with 2′,7′-dichlorofluorescin diacetate. Each experiment was repeated three times. Data are expressed as mean values ± SEM and were analyzed by *t*-test. *** *p* < 0.001 vs. ctrl (control).

Neutrophil oxidative burst has been reported to promote tumor progression [17]. As shown in Figure 3a, an increase in intracellular ROS levels was detected in dHL60 cells treated with A375 CSC–CM. As expected, this was paralleled by an enhanced production of extracellular H_2_O_2_ (Figure 3b). Collectively, these results highlight once again the ability of melanoma SCs to confer N2 traits to neutrophils.

### 3.4. Melanoma SC–CM Induces NET Release from Neutrophils in a Cell Death-Independent Manner

**Figure 4:**
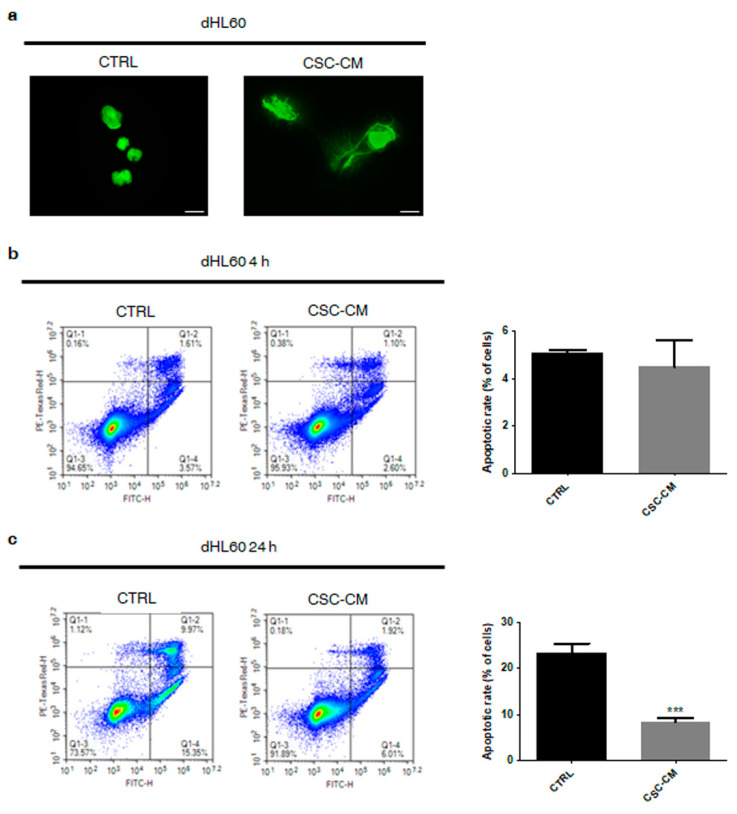
Melanoma SC–CM induces NET release from neutrophils in a cell death-independent manner. (**a**) dHL60 cells were incubated with CSC–CM for 4 h. NET release was then evaluated by SITOX Green assay. One representative experiment from three independent tests is shown. Scale bars are 20 μm. (**b**) dHL60 cells were incubated with CSC–CM for 4 h. Apoptotic rates were then evaluated by flow cytometry after staining with eBioscience™ Annexin V-FITC Apoptosis Detection Kit. Each experiment was repeated three times. Data are expressed as mean values ± SEM and were analyzed by *t*-test. (**c**) dHL60 cells were incubated with CSC–CM for 24 h. Apoptotic rates were then evaluated by flow cytometry after staining with eBioscience™ Annexin V-FITC Apoptosis Detection Kit. Each experiment was repeated three times. Data are expressed as mean values ± SEM and were analyzed by *t*-test. *** *p* < 0.001 vs. ctrl (control).

The release of neutrophil extracellular traps (NETs) is another well-known hallmark of the N2 phenotype [18,19]. As shown in Figure 4a, it was largely observed in dHL60 cells exposed to A375 SC–CM but not in controls. Intriguingly, this was not accompanied by the induction of cell death, demonstrating that NET secretion does not correlate with suicidal NETosis (Figure 4b); on the contrary, a significant increase in cell survival was found in CSC–CM-incubated immune cells, evidencing the ability of melanoma SCs to protect neutrophils from spontaneous apoptosis (Figure 4c).

### 3.5. Melanoma SC-Activated Neutrophils Secrete Increased Levels of Tumor-Promoting Factors

**Figure 5:**
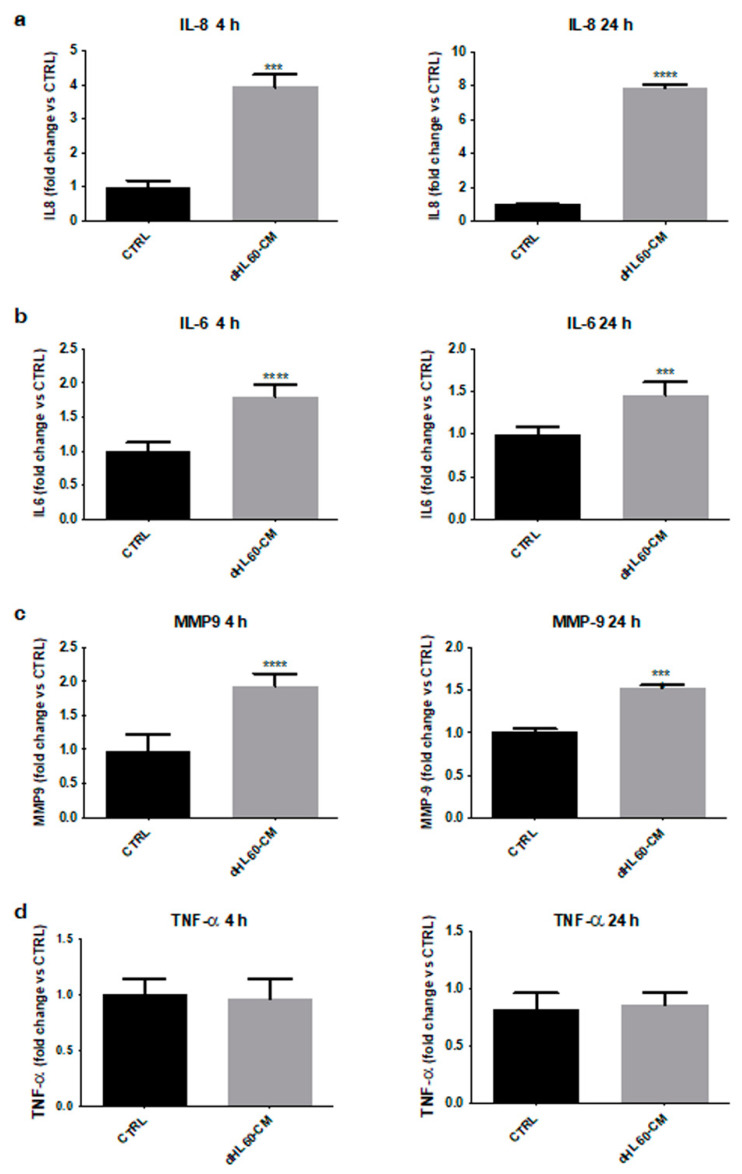
Melanoma SC-activated neutrophils secrete increased levels of tumor-promoting factors. (**a**) dHL60 cells were incubated with CSC-CM for 4 and 24 h, then their supernatant was changed with proper medium for 24 h. IL-8 secretion was then assessed by Elisa assay. Each experiment was repeated three times. Data are expressed as mean values ± SEM and were analyzed by *t*-test. *** *p* < 0.001 vs. ctrl (control), **** *p* < 0.0001 vs. ctrl (control). (**b**) dHL60 cells were incubated with CSC–CM for 4 and 24 h, then their supernatant was changed with proper medium for 24 h. IL-6 secretion was then assessed by Elisa assay. Each experiment was repeated three times. Data are expressed as mean values ± SEM and were analyzed by *t*-test. *** *p* < 0.001 vs. ctrl (control), **** *p* < 0.0001 vs. ctrl (control). (**c**) dHL60 cells were incubated with CSC–CM for 4 and 24 h, then their supernatant was changed with proper medium for 24 h. MMP-9 secretion was then assessed by Elisa assay. Each experiment was repeated three times. Data are expressed as mean values ± SEM and were analyzed by *t*-test. *** *p* < 0.001 vs. ctrl (control), **** *p* < 0.0001 vs. ctrl (control). (**d**) dHL60 cells were incubated with CSC–CM for 4 and 24 h, then their supernatant was changed with proper medium for 24 h. TNF-α secretion was then assessed by Elisa assay. Each experiment was repeated three times. Data are expressed as mean values ± SEM and were analyzed by *t*-test.

To further explore the role of melanoma SCs in neutrophil polarization, the immune cell secretome was analyzed. Figure 5a–c highlights an enhanced production of different tumor-sustaining factors, such as IL-8, IL-6, and MMP-9, in dHL60 cells activated with A375 CSC–CM; on the other hand, no significant changes in the synthesis of TNF-α, a well-known N1 state marker, were observed (Figure 5d). These data confirm that treatment with melanoma SC–CM polarizes neutrophils towards an N2 phenotype.

### 3.6. Melanoma SC-Incubated Neutrophils Confer Stemness Properties to Melanoma Non-Stem Cells

**Figure 6:**
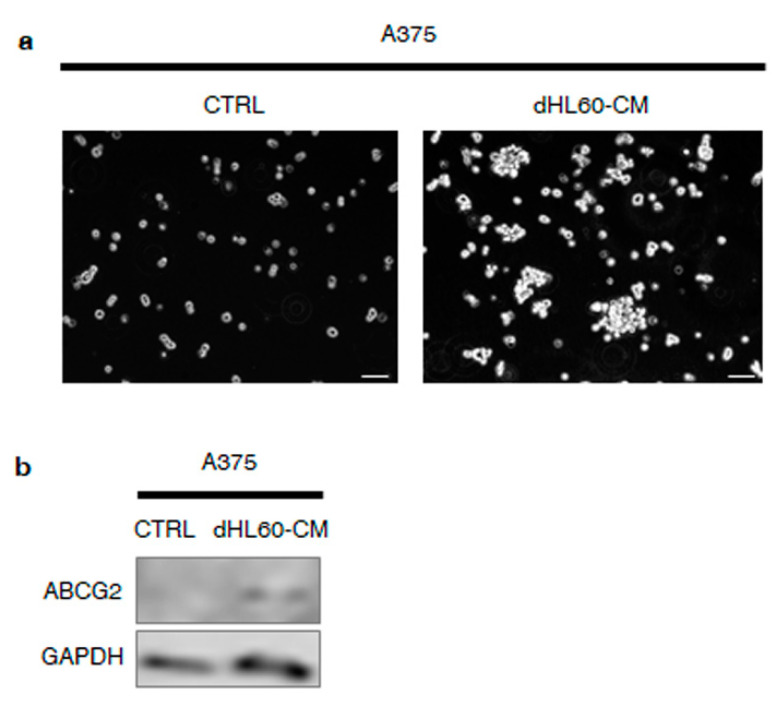
Melanoma SC-incubated neutrophils confer stemness properties to melanoma non-stem cells. (**a**) A375 cells were incubated with dHL60–CM, obtained from dHL60 cells previously exposed to CSC–CM for 24 h. After 7 days, sphere formation assay was performed to determine the spheroidogenic ability of A375 cells. One representative experiment from three independent tests is shown. Scale bars are 30 μm. (**b**) A375 cells were incubated with dHL60–CM, obtained from dHL60 cells previously exposed to CSC–CM for 24 h. After 7 days, Western blot analysis was carried out to analyze the expression of ABCG2. GAPDH was used as a loading control. One representative experiment from three independent tests is shown.

The above evidence suggests that neutrophils exposed to melanoma SC–CM may be deeply implicated in tumor progression. To test this hypothesis, we treated A375 cell line with the CM from dHL60 cells previously exposed to CSC–CM. As reported in Figure 6a, this was followed by higher sphere formation rates. In addition, an upregulation of the stemness marker ABCG2 was found (Figure 6b). Taken together, these findings demonstrate that melanoma SC-incubated neutrophils are able to confer stemness features to melanoma non-stem cells.

## 4. Discussion

It is now widely accepted that CSCs are deeply involved in tumor initiation and evolution. In particular, a growing body of evidence points to a key role of this small cell subpopulation in regulating the interactions with the immune TME, particularly with neutrophils. For instance, colorectal cancer SCs have been recently found to release extracellular vesicles able to promote the switch towards an N2 phenotype in immune cells [10]. Similarly, a study by Vashendriya et al. has revealed the importance of neutrophils in driving glioma stem-like cell homing to the metastatic niche [11]. However, investigations have only begun to elucidate the relationship between CSCs and the immune system. Here, we dissected the molecular mechanisms underlying the bidirectional crosstalk occurring between melanoma SCs and neutrophils. 

First, we demonstrated that A375 CSCs can directly activate dHL60 immune cells via upregulation of common surface markers, such as CD66b and CD11b. In particular, they were found to stimulate neutrophil chemotaxis by producing various cytokines implicated in their recruitment and phenotypic switch from N1 to N2, including TGF-β, IL-6, and IL-8. This correlated with STAT3 and ERK1/2 phosphorylation, CXCR2/NF-kB overexpression, and P38 activation in dHL60 cell line. These data are consistent with previous reports describing the crucial role of the above pathways in neutrophil transition towards a tumor-promoting state [6,20,21]. Indeed, it has been widely observed that inhibition of TGF-β secretion in the TME results in the recruitment of neutrophils with an N1 phenotype [22]. On the other hand, deregulation of the IL-6-STAT3-ERK-1/2 cascade correlates with N2 polarization of immune cells in liver and gastric cancer [23,24]. Similarly, IL-8-CXCR2 interaction has been reported to favor the migration of cancer-associated neutrophils towards the pancreatic carcinoma metastatic niche, and IL-8-mediated increase in NF-kB expression leads to the acquisition of a pro-tumor immune profile [25,26]. Finally, P38 signaling has recently emerged as a crucial modulator of neutrophil chemotaxis and tumor-sustaining activities [14,15,16]. Our results not only confirm this evidence, but also highlight the involvement of a complex tumor–stroma interaction network in melanoma onset and progression.

ROS overproduction and NET release are well-known hallmarks of N2-polarized neutrophils. Indeed, alterations of the redox homeostasis in the TME have been linked to the inhibition of T-cell response to cancer cells [27]. Likewise, it has been recently proposed that NETs can act as a physical barrier between tumor cells and CD8+ T and natural killer (NK) cells, protecting the first from the cytotoxicity of the latter [28]. In our study, we analyzed these mechanisms in A375 CSC–CM-treated dHL60 neutrophils, highlighting the induction of both processes in these cells. Remarkably, no parallel cell death was observed; in contrast, an increased cell lifespan was evidenced, supporting the ability of melanoma SCs to drive a long-term pro-tumor activation in neutrophils.

Several studies have shown that cancer-associated neutrophils are characterized by the secretion of various pro-tumor factors [29,30,31,32]. Herein, an enrichment in IL-6, IL-8, and MMP-9 levels was found in the supernatant of A375 CSC-primed dHL60 cells. This is in agreement with recent findings evidencing the contribution of these proteins in the emergence of an immunosuppressive TME [33,34,35], and it further validates the fundamental role played by melanoma SC subpopulation in the education of the immune system.

To finally verify the hypothesis of a dual dialogue between melanoma SCs and neutrophils, we investigated the effects of the medium from CSC–CM-exposed dHL60 cell line on A375 cells. Remarkably, we revealed that N2-polarized neutrophils could confer stem-like traits to melanoma cells, by promoting their spheroidogenic ability as well as the expression of ABCG2. In this regard, emerging evidence points to a key role of the immune system in driving cancer stemness in ovarian, prostate, and colorectal cancer [36,37,38]. To our knowledge, this is the first study highlighting the role of cancer-associated neutrophils in promoting melanoma progression.

## 5. Conclusions

Immune escape represents a major step in carcinogenesis and metastasis, with several studies reporting the ability of cancers to remodel the TME and evade the innate surveillance of a wide range of cell types, including macrophages, microglia, NK cells, and, above all, neutrophils [39,40,41,42,43]. Although considerable progress has been made in understanding how tumors elude destructive immunity, the function of CSCs in this process is still unclear. Our findings demonstrate that melanoma SCs are able to prime neutrophils towards a pro-tumor N2 phenotype, which in turn endow cancer cells with stemness properties.

## Data Availability

The data presented in this study are available on request from the corresponding author.

## Supplementary Materials

The following supporting information can be downloaded at: https://www.mdpi.com/article/10.3390/cancers14143391/s1, Supplementary figure: The original WB of Figure 2 and Figure 6.
