# Supplementary figures and images for: Melanoma Stem Cells Educate Neutrophils to Support Cancer Progression
Source: Cancers (Basel). 2022 Jul 13;14(14):3391. doi: 10.3390/cancers14143391 (PMC9317939; doi:10.3390/cancers14143391)

P-ERK

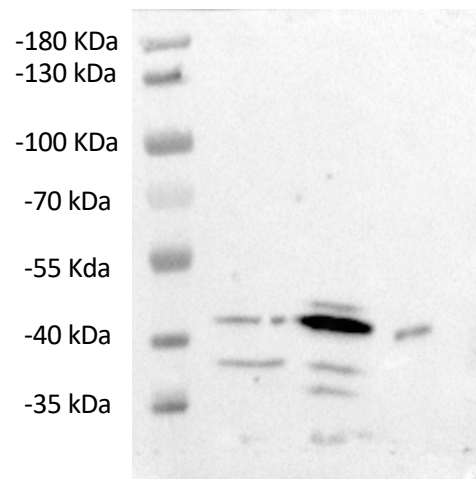

ERK

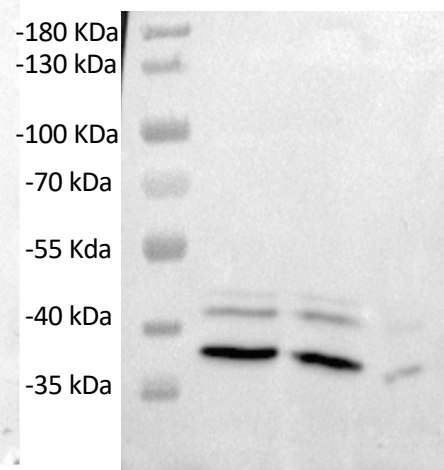

GAPDH

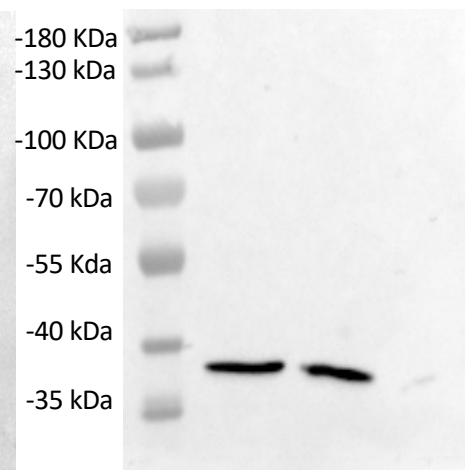

P-STAT3

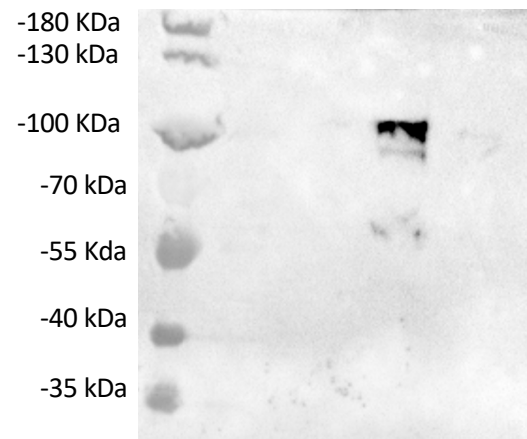

STAT3

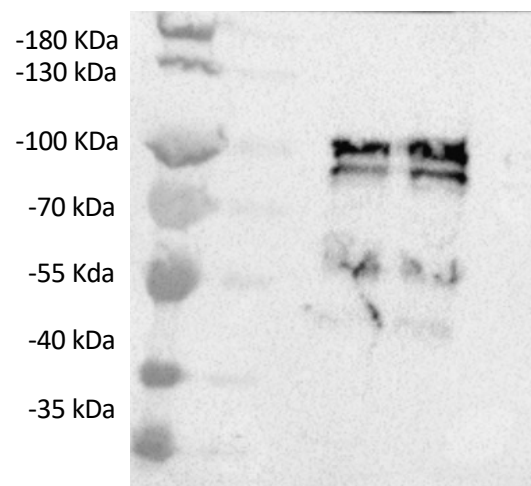

NF-kB

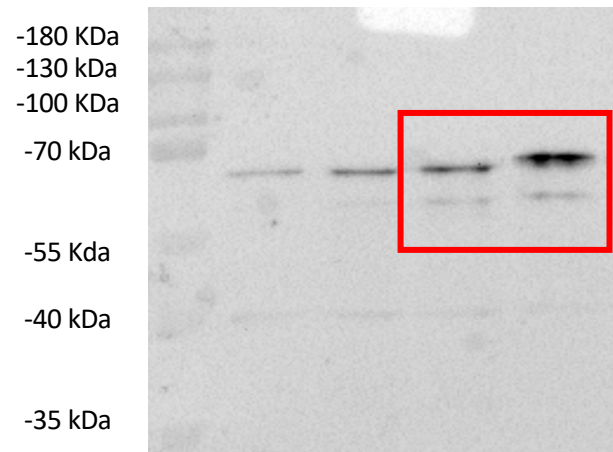

GAPDH

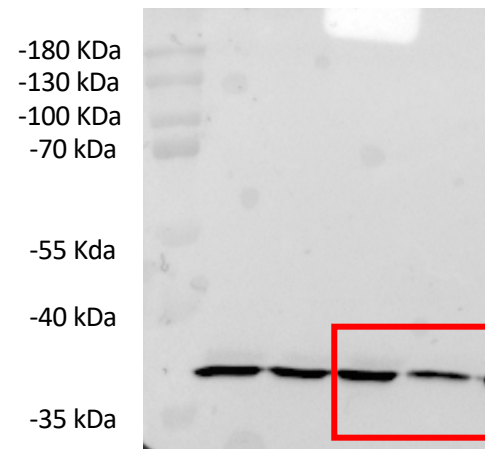

ABCG-2

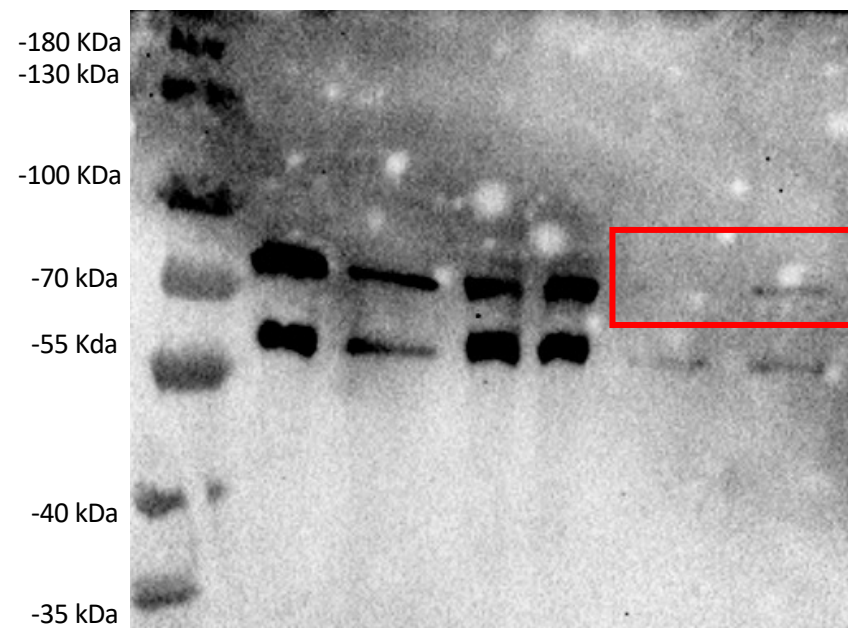

GAPDH

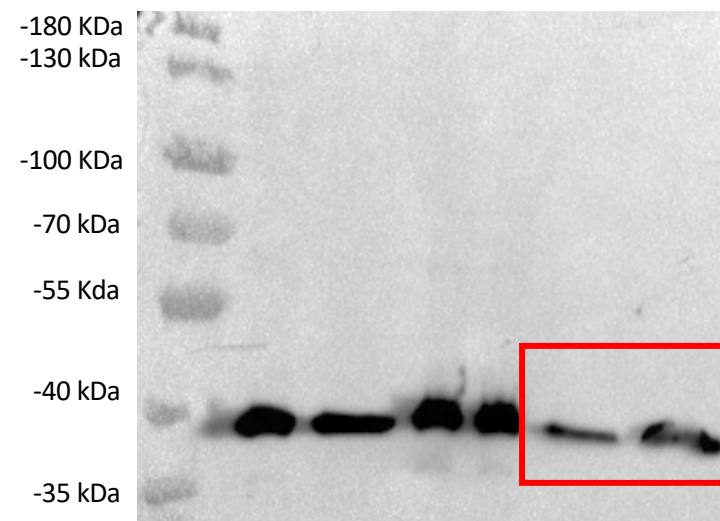

Supplement: Supplementary file 1 [file cancers-14-03391-s001.zip › cancers-1793980-supplementary.pdf]
